# Prevalence and Socio-Demographic Correlates of Body Weight Categories Among South African Women of Reproductive Age: A Cross-Sectional Study

**DOI:** 10.3389/fpubh.2021.715956

**Published:** 2021-10-25

**Authors:** Monica Ewomazino Akokuwebe, Erhabor Sunday Idemudia

**Affiliations:** Faculty of Humanities, North West University, Mafikeng, South Africa

**Keywords:** body weight categories, nutrition transition, prevalence, South Africa, socio-demographic, women of reproductive age

## Abstract

**Background:** The shift in disease patterns has been connected with increased body weight burden, becoming a major public health concern in South Africa, as previous studies have assessed overweight or obesity among certain populations. However, little is known about bodyweight burden (underweight, overweight, and obesity) among women aged 15–49 years. Therefore, this study was conducted to identify the prevalence and its associated socio-demographic correlates of bodyweight categories among women of reproductive age in South Africa.

**Methods:** The present study used the South Africa Demographic Health Survey (2016 SADHS) data for 2016. A total of 3,263 women of reproductive age were included in the analysis. Both bivariable and multivariable logistics regressions were performed to determine the prevalence and socio-demographic correlates of bodyweight categories among women in South Africa. Thus, this study used the criteria of the WHO standard body mass index (BMI) cut-offs to classify bodyweight categories. The odds ratios (ORs) with 95% CIs were estimated for potential determinants included in the final model.

**Results:** The overall prevalence of body weight burden was 66.5%, with 4.9% underweight, 27.1% overweight, and 34.5% obese (*p* < 0.05). The identified factors associated with underweight among women of reproductive age were those from “other” population group [adjusted odds ratio (AOR) 2.65: 95% CI 1.40–5.00], rural residence (AOR 1.23: 95% CI 0.75–2.02), and Northern Cape Province (AOR 1.58: 95% CI 0.65–3.87). For overweight/obese, the main factors were those aged 45–49 years (AOR 10.73: 95% CI 7.41–15.52), tertiary education (AOR 1.41: 95% CI 0.97–2.03), and residing in Eastern Cape (AOR 1.27: 95% CI 0.82–1.99) and KwaZulu-Natal Provinces (AOR 1.20: 95% CI 0.78–1.84).

**Conclusion:** The findings presented in this study indicate the concurrence of underweight and overweight/obese among women aged 15–49 years in South Africa. Despite underweight prevalence being on the decline, yet overweight/obese is increasing over time. The health implication of body weight burden needs rapid and effective interventions, focusing on factors such as rural, education, population group, older age 45–49 years, and Provinces (Northern Cape, Eastern Cape, and KwaZulu-Natal) – the high-risk groups identified herein are of most importance to curb the growing burden among South African women of reproductive age.

## Background

The population dynamics of the rapid and major demographic transitions associated with socio-economic development and accompanied by an epidemiological transition have emerged in industrialised countries and across sub-Saharan African (SSA) countries. The emergence of demographic and epidemiological transitions has led to a decrease in widely known acute infectious diseases and a rising prevalence of non-communicable diseases (NCDs) and chronic degenerative diseases (1, 2). This increased burden of disease and the double burden of malnutrition (undernutrition and over-nutrition) in low-income countries have become a public health concern, which has received attention from both health and non-health experts. With increased knowledge of demographic changes and their impact on the nutrition of the general population, the shift in disease patterns towards diet- or nutrition-related NCDs has been linked with behavioural/lifestyles changes, diets, and environmental exposure. In effect, it has continued to cause a gradual shift in the age pattern of NCD mortality among younger persons (<60 years old) more than those in older age cohorts (>60 years old) (3, 4). However, the reasons for these increasing trends of the age pattern of NCDs are not completely understood.

Several studies have indicated a high prevalence of over-nutrition, which has increased by more than 33.0% (5, 6), contributing to a rapid rise in the NCD burdens in Africa (2, 7). The yearly contributions of each of the major NCD-related deaths include cardiovascular diseases account for 17 million deaths, cancers account for 7.6 million deaths, respiratory diseases account for 4.2 million deaths, and diabetes accounts for 1.3 million deaths (8). Other largely known risk factors shared by these four diseases include tobacco use, physical inactivity, harmful use of alcohol, and an unhealthy diet (9, 10). The occurrence of overweight and obesity has steadily been stated to have intensified, albeit with variation, in developed and developing countries across homogeneous and heterogeneous populations due to a prevalent “obesogenic” environment (11, 12). The contributing factors of obesity need to be better understood as the aetiology of obesity is complex (13, 14), despite the fact that globalisation and urbanisation are two of the key drivers of the malnutrition endemic in South Africa. In addition, to the physiological anatomy of individuals (15), there are behavioural/lifestyle determinants (16) along with economic (17) and environmental/socio-cultural factors (18). Thus, these factors either directly or indirectly have an influence on overweight and obesity progression among women in South Africa.

Since 1940, South Africa has been undergoing a nutrition transition, with an increase in the contribution of fats and a decrease in carbohydrates and fibre intake towards energy consumption (13, 14). Thus, nutrition transition was found to be faster among the black racial groups than the white and Indian/Asian racial groups (19, 20) and in urban rather than rural populations (21), driving an upsurge in obesity in the black population. Pertaining to provinces, for instance, Eastern Cape is the third largest province and the second poorest in South Africa, where 50% of households in the rural districts are food insecure (22). However, studies on the incidence of overweight and obesity in these rural areas are scarcely documented.

Replicating consistent research outcomes of socio-demographic and behavioural/lifestyles risk factors by body weight in developed nations, these and other studies have created indication of a connexion of body mass index (BMI) with gender and with behavioural risk factors: higher BMI among women than men, alcohol ingestion (positive relationship), tobacco use (negative relationship), physical exercise (higher level of physical body exercise associated with lower BMI), and place of residence (urban vs. rural living, with the former associated with higher values of BMI). Considering the observed trends in the BMI, distributions of risk factors (such as increasing urbanisation and average socioeconomic status, stable but high alcohol intake, reduction in tobacco smoking, and decrease physical body exercise) in the population have not been given full attention in non-medical studies (16, 23); yet there is an indication that BMI is connected with factors such socioeconomic status, which is also comparatively proven in demographic and population studies. However, unlike what has been observed in developed countries, there exists an assumption that observed variations in BMI and the prevalence of obesity in South Africa are at least partly driven by changes in the distribution of the above-mentioned risk factors.

Notably, these determinants appear to be remarkably different across culture, age, gender, and social class (24). In addition, there are gaps in knowledge regarding socio-cultural determinants of underweight and overweight/obesity, in particular, at the national level (13, 14). Thus, this study set out to explore the prevalence and socio-demographic correlates of the bodyweight of women in South Africa. Moreover, being underweight or overweight is likely to lead to adverse health outcomes throughout life (25), such as the increased risk of maternal disease and adverse pregnancy outcomes (26). The upsurge in the prevalence of obesity at its 2010 level and the worldwide NCD target of halting obesity by the year 2025 have been driven mostly by health concerns and the economic burden of an increasing BMI (27, 28). In addition, there is a realisation from the previous studies that there are scarce analyses on the trends of underweight (23), particularly for the male populations. Disparities in the levels of underweight and obesity across African regions, for example, in Southern African countries comprising Botswana, Namibia, South Africa, and Swaziland (now Eswatini), the obesity index is highest (13, 21, 29–32). In the year 2008, South Africa was rated with the highest BMI, with a median score at the population level approximated at 26.9 kg/m^2^ among males (in contrast to a world average of 23.8 kg/m^2^), and 29.5 kg/m^2^ among females (in contrast to a world average of 24.1 kg/m^2^), respectively.

It is important for respective nations to identify their predictable prevalence and changes in body weight (underweight, overweight, and obese) so that this can be used as a basis by governmental stakeholders to improve and enforce suitable intervention policies. At the moment, South Africa does not have ample evidence on the prevalence and its associated socio-demographic correlates of bodyweight categories (underweight, normal, overweight, and obese) among women of reproductive age. Consequently, the research questions under investigation in the current study are: what is the prevalence of each bodyweight category and what are the associated socio-demographic correlates of body weight of women of reproductive age? To avert a further health burden of unhealthy bodyweight categories in the general population of South Africa, it is important that the prevalence, change over time, and communal factors that are quantifiable and responsive to intervention should be identified. We utilised nationally representative data collected using similar methods and techniques to determine the prevalence and correlates of bodyweight categories associated with socio-demographic factors among women of reproductive age in South Africa. This study will, therefore, add valuable information in describing the prevalence of body weight associated with socio-demographic factors to identify immediate and effective interventions for South African women with problems of body weight.

## Methods

### Data Source

This study utilised the data from the third round of the South Africa Demographic and Health Survey, conducted in 2016 (2016 SADHS). The 2016 SADHS is a nationally representative sample survey of 15,292 households; 8,514 eligible women in the age range 15–49 years were interviewed with a response rate of 86% (32). The primary objective of this survey was to provide updated and reliable information on marriage and sexual activity, fertility, fertility preferences, contraception, infant and child mortality, maternal healthcare, child health, nutrition of children, HIV/AIDS-related knowledge and behaviour, HIV prevalence, adult and pregnancy-related mortality, use of health services and prescribed medications, adult morbidity, adult nutrition, tobacco, alcohol, and codeine use among adults, women's empowerment, and domestic violence. A stratified two-stage design was utilised in sample selection comprising 750 primary sampling units (PSUs); 468 in urban, 224 in rural, and 58 from farm areas, from a list provided by Statistics South Africa (Stats SA). In stage one, PSUs were selected using probability proportional to PSU size. In stage two, 220 residential dwelling units (DUs) from each PSU were selected with an equal chance of systematic selection from the household listing. The sampling frame used for the 2016 SADHS was the 2011 South African Population and Housing Census. The 2016 SADHS covered age groups of 15–49 years, i.e., those of reproductive age, which made it possible to identify the prevalence of the outcome and explanatory variables associated with weight status. Since the data used in the survey represent only the participants sampled, the data were weighted to make it nationally representative of the participants aged 15–49 years. A comprehensive report of the sampling techniques is provided in the national report of 2016 SADHS (32). This study is based on 5,251 women of reproductive age (15–49 years) who had at least one live birth in the past 5 years preceding the survey. However, 1,988 women who did not respond to BMI questions and who were pregnant at the time of the survey were excluded from the analysis, and a total of 3,263 women were included in the final analysis.

### Description of the Measurement of BMI and Its Classification

During the 2016 SADHS, field workers used the portable height/length board in measuring height in centimetres, which was later converted to metres, with restrictions to 1.0–2.7 m (32). Weights were measured using Seca 213 portable stadiometers and formed the boundary of 20–350 kg as advocated by the WHO (33, 34). Using these boundaries, 1.1% of the respondents were in exception in 2016. BMI is calculated with the metric system as follows:


$$\text{BMI}=\frac{\text{Person's weight}\left(\text{kg}\right)}{\text{Person's height}{\left(\text{m}\right)}^{2}}$$

(where kg is kilogrammes and m is metres).

Note that height is commonly measured in centimetres (cm), and height (cm) is divided by 100 to obtain height in metres (m). With the WHO recommendations, the standard weight status categories associated with BMI ranges for adult men and women are the same for all body types and ages. Thus, epidemiological studies have shown substantial risk in people with very high BMI, for instance, severe (≥35 kg/m^2^) or morbid (≥40 kg/m^2^) obesity (10).

### Outcome Variables

The outcome variable for this study was BMI, which was dichotomized as underweight and overweight/obese, respectively. In view of this, binary outcomes with two possible values were constructed as the dependent variable for this study based on underweight vs. normal weight and overweight/obese vs. normal weight, respectively, based on the WHO standard BMI cut-offs (33, 34). Thus, women who were underweight or overweight/obese were coded “1,” and those with normal weight were coded “0.” This categorisation was done to ensure large sample sizes for analyses and to obtain more robust binary logistic regression estimates (35–37). Women with BMI < 18.5 kg/m^2^ were described as underweight, while those with BMI of 18.5–24.9 kg/m^2^ were described as having normal body weight, those with BMI of ≥25 kg/m^2^ were overweight, and those with BMI ≥ 30 kg/m^2^ were obese.

### Explanatory Variables

The selected socio-demographic predictor factors incorporated in the analysis are age (15–19, 20–24, 25–29, 30–34, 35–39, 40–44, and 45–49), marital status (never married and ever married), population group (African/Black and other), educational level (not educated and educated), place of residence (urban and rural), work status (not employed and employed), provinces (Western Cape, Eastern Cape, Northern Cape, Free State, KwaZulu-Natal, North West, Gauteng, Mpumalanga, and Limpopo), and wealth quintile (lowest, middle, and highest). However, the household wealth quintile is a substitute for household economic status and was assessed from possession of household assets, such as consumer items and dwelling characteristics. A score was created for each individual using principal component analysis and classified into five quintiles as Lowest (Poorest), Second (Poorer), Third (Middle), Fourth (Rich), and Highest (Richest) (32).

### Statistical Analysis

The data were weighted using sample weights, and the weighted data were used to study the characteristics of the respondents, adjusted for the degree of differences of odds of selection, as the sample design involves more than one stage of selection. This further ensured that data were representative of the target population; in this case, women aged 15–49 years old. To identify the prevalence and socio-demographic correlates of body weight, statistical analyses were carried out at the univariate, bivariate, and multivariate levels. At the univariate level, frequencies and percentages were used to describe the study population and bar graphs was used to describe the prevalence of body weight categories among women (aged 15–49 years). The bivariate analyses were carried out to examine the nature of the association between body weights by selected socio-demographic characteristics. Also at the multivariate level, binary logistic regressions were employed to assess the socio-demographic determinants of body weight of women. The binary regression was calculated as the exponential function of the regression coefficient (e^b1^) as the measure of the odds ratios (ORs) associated with the outcome and explanatory variables. The findings from the regression analysis were presented as an unadjusted odds ratio (UOR) and adjusted odds ratio (AOR), using 95% CIs and sample covariates (socio-demographic factors) were used to estimate the outcomes. All analyses performed were carried out using STATA version 12.1 (StataCorp LP, College Station, TX, USA).

### Ethical Statement

The current analysis is based on the use of secondary datasets from the 2016 SADHS. The 2016 SADHS was conducted under the scientific and administrative supervision of Stats SA, in partnership with the South African Medical Research Council (SAMRC), which conducted the 2016 SADHS, at the request of the National Department of Health (NDoH). Stat SA performed an independent Ethics review of the 2016 SADHS protocol. The data collection procedures were also monitored and approved by the ICF Macro DHS programme team, Calverton, MA, USA. All individuals selected in the SADHS were provided with informed voluntary and written consent. Approval of the individual was sought, and only then was the interview conducted. The survey data collection took place from June 27, 2016 to November 4, 2016. The SADHS dataset is in the public domain and accessible upon a request granted from the Demography Health Survey (DHS) programme (http://www.measuredhs.com).

## Results

### Socio-Demographic Characteristics of Study Respondents

Table 1 depicts the socio-demographic characteristics of the respondents. The majority of women were in the age cohorts of 15–29 years (50.6%), and 62.2% were never married. More than two-thirds of the women were African/Black, and most of the respondents had secondary education (78%). The majority of women were residing in urban areas, belonged to the unemployed category (70%), the lowest wealth quintile (43.2%), and were from the KwaZulu-Natal province of the country.

**Table 1:** Socio-demographic characteristics of the weighted samples of women aged 15–49 years old in South Africa, 2016.

**Variables**	**%**	***n***
**Age**		
15–19	17.3	563
20–24	16.3	532
25–29	17.0	554
30–34	14.3	467
35–39	12.7	415
40–44	11.5	375
45–49	10.9	357
**Marital status**		
Never married	62.2	2,030
Ever married	37.8	1,233
**Population group**		
African/Black	89.2	2,911
Other	10.8	352
**Educational level**		
No education and primary	13.1	427
Secondary	77.9	2,542
Higher	9.0	294
**Residence**		
Urban	53.5	1,747
Rural	46.5	1,516
**Work status**		
Not employed	69.9	2,281
Employed	30.1	982
**Wealth quintile**		
Lowest	43.2	1,411
Middle	24.5	800
Highest	32.2	1,052
**Provinces**		
Western Cape	5.1	166
Eastern Cape	13.0	425
Northern Cape	9.1	297
Free State	10.4	338
KwaZulu-Natal	15.9	520
North West	10.6	346
Gauteng	9.2	299
Mpumalanga	13.3	434
Limpopo	13.4	438
**Total**, ***n***	**100.0**	**3,263**

*South Africa Demographic Health Survey (SADHS) (32)*.

### Percentage Prevalence of Bodyweight Categories Among Women Aged 15–49 Years

**Figure 1:**
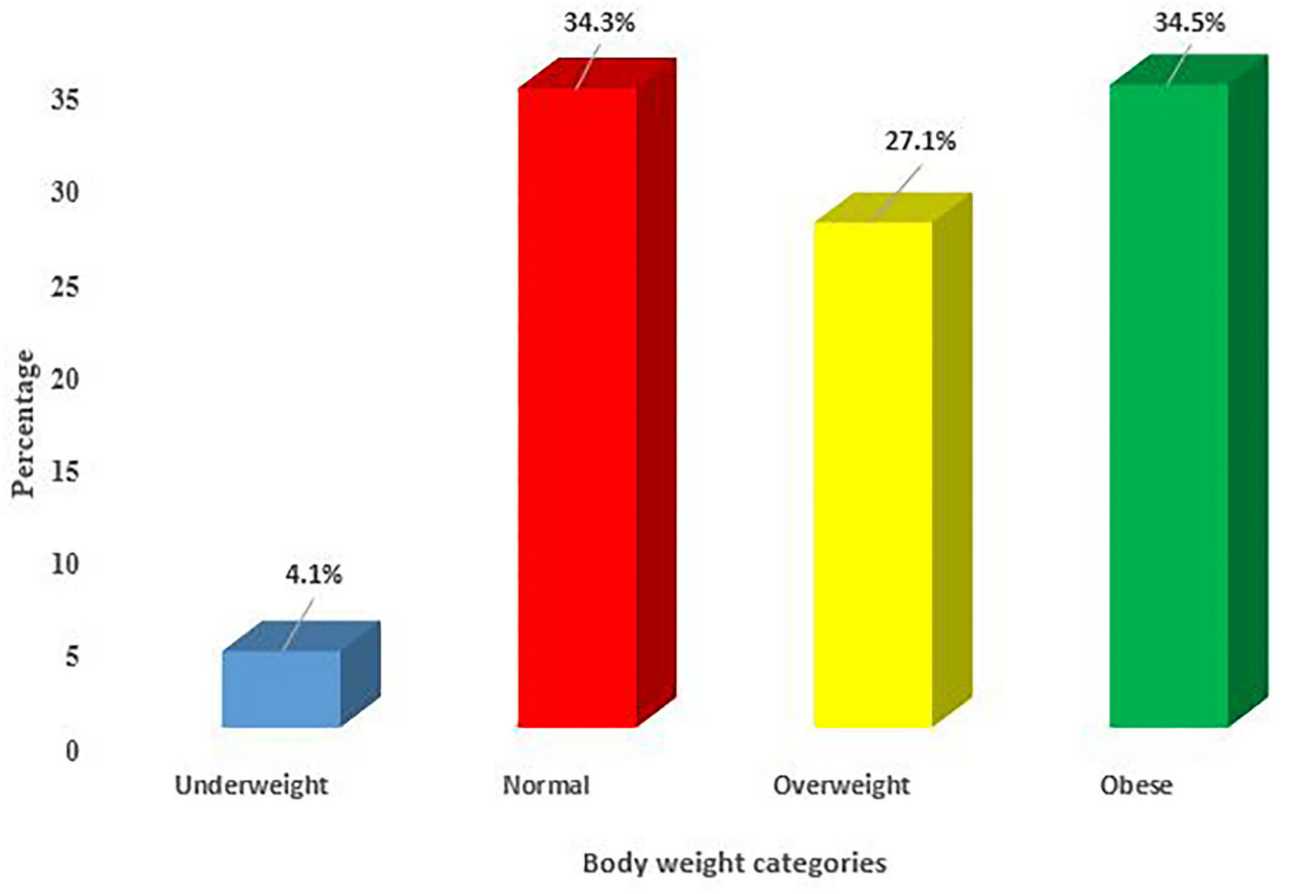
Percentage prevalence of bodyweight categories among women aged 15–49 years who had at least one live birth in the past 5 years preceding the survey in South Africa, 2016.

Figure 1 illustrates the four body weight categories of nutrition among women of reproductive age in South Africa. The bar chart reveals that a majority of the women aged 15–49 years were obese (34.5%) followed by those in the normal body weight category (34.3%) in South Africa (Figure 1).

Table 2 presents the Chi-square results of the body weight by socio-demographic of women aged 15–49 years in South Africa. Socio-demographic factors such as age, marital status, population group, education, work status, and province have significant relationships with underweight. The prevalence of underweight is 4.1%, and overall, underweight is more prevalent among women who are never married (5.7%). The proportion of underweight women among Black/African is lower than the other population groups (7.4%), and 4.3% of women with secondary education are underweight compared with women with no education/primary, and higher educational level (Table 2). Rural women (4.4%) are more likely to be underweight than their urban counterparts (3.9%). There is a significant association among women who are not employed. About 4.6% of women who are underweight are in the middle wealth quintile, and among women residing in Northern Cape Province, 8.8% are underweight (Table 2).

**Table 2:** Chi-square associations of body weight by socio-demographic factors among weighted samples of women aged 15–49 years in South Africa, 2016.

**Socio-demographic factors**	**2016**					
	**Underweight**			**Overweight and obese**		
	**No**	**Yes**	***p*-value**	**No**	**Yes**	***p*-value**
**Age**			**0.000**			**0.000**
15–19	506 (89.9)	57 (10.1)		404 (71.8)	159 (28.2)	
20–24	506 (95.1)	26 (4.9)		255 (47.9)	277 (52.1)	
25–29	536 (96.7)	18 (3.3)		210 (37.9)	344 (62.1)	
30–34	455 (97.4)	12 (2.6)		134 (28.7)	333 (71.3)	
35–39	401 (96.6)	24 (3.7)		104 (25.1)	311 (74.9)	
40–44	371 (98.9)	4 (1.1)		87 (23.2)	288 (76.8)	
45–49	353 (98.9)	4 (1.1)		59 (16.5)	298 (83.5)	
**Marital status**			**0.000**			**0.000**
Never married	1,915 (94.3)	115 (5.7)		945 (46.6)	1,085 (53.4)	
Ever married	1,213 (98.4)	20 (1.6)		308 (25.0)	925 (75.0)	
**Population group**			**0.000**			**0.575**
Black/African	2,802 (96.3)	109 (3.7)		1,113 (38.2)	1,798 (61.8)	
Other	326 (92.6)	26 (7.4)		140 (39.8)	212 (60.2)	
**Educational level**			**0.000**			**0.010**
No education and primary	411 (96.3)	16 (3.7)		171 (40.1)	256 (59.9)	
Secondary	2,433 (95.7)	109 (4.3)		993 (39.1)	1,549 (60.9)	
Higher	284 (96.6)	10 (3.4)		89 (30.3)	205 (69.7)	
**Residence**			**0.451**			**0.031**
Urban	1,679 (96.1)	68 (3.9)		641 (36.7)	1,106 (63.3)	
Rural	1,449 (95.6)	67 (4.4)		612 (40.4)	904 (59.6)	
**Work status**			**0.000**			**0.000**
Not employed	2,167 (95.0)	114 (5.0)		1,008 (44.2)	1,273 (55.8)	
Employed	961 (97.9)	21 (2.1)		245 (24.9)	737 (75.1)	
**Wealth quintile**			**0.444**			**0.000**
Lowest	1.350 (95.7)	61 (4.3)		619 (43.9)	792 (56.1)	
Middle	763 (95.4)	37 (4.6)		289 (36.1)	511 (63.9)	
Highest	1,015 (96.5)	37 (3.5)		345 (32.8)	707 (67.2)	
**Provinces**			**0.000**			**0.116**
Western Cape	159 (95.8)	7 (4.2)		55 (33.1)	111 (66.9)	
Eastern Cape	416 (97.9)	9 (2.2)		145 (34.1)	280 (65.9)	
Northern Cape	271 (91.2)	26 (8.8)		130 (43.8)	167 (56.2)	
Free State	321 (95.0)	17 (5.0)		125 (37.0)	213 (63.0)	
KwaZulu-Natal	510 (98.1)	10 (1.9)		193 (37.1)	327 (62.9)	
North West	330 (95.4)	16 (4.6)		130 (37.6)	216 (62.4)	
Gauteng	292 (97.7)	7 (2.3)		115 (38.5)	184 (61.5)	
Mpumalanga	414 (95.4)	20 (4.6)		176 (40.6)	258 (59.4)	
Limpopo	415 (94.7)	23 (5.3)		184 (42.0)	254 (58.0)	

*p < 0.001, p < 0.01, p < 0.05 is considered statistically significant (Chi-Square test)*.

In addition, Table 2 presents the Chi-square results of overweight/obese by socio-demographics of women in South Africa. The bivariate analysis shows that age, marital status, education, place of residence, work status, and wealth quintile have a significant association with overweight/obese. From the table, the findings revealed that women aged 45–49 years were found to be more overweight/obese (83.5%) than other age cohorts; while 75% of those ever married were found to be overweight/obese, while 61.8% were overweight/obese among Black/African population group. Furthermore, 69.7% of women with higher education were overweight/obese. For women living in urban areas, 63.3% of them were overweight/obese, and 75.1% of the employed women were overweight/obese. Women in the highest wealth quintile were more overweight/obese, and women residing in Western Cape were found to be more overweight/obese (66.9%; Table 2).

Table 3 presents multivariate logistic regressions of the UOR and AOR on underweight and overweight/obese and their socio-demographic factors in reference to normal weight among women aged 15–49 years. Thus, compared with women aged 15–19 years, women within the age cohort of 20–24 years were 0.46 times less likely to be underweight. Women in the age cohort of 20–24 years were 2.76 times more likely to be overweight/obese compared to women who were 15–19 years old. Adjusting for other variables used in this study, the findings indicated that women who were 40–44 years old were 0.1 times less likely to be underweight compared to their counterparts who are in the age cohort of 15–19 years. The 40–44-year-old women were found to be 6.79 times more likely to be overweight/obese compared with those aged 15–19 years. In addition, the odds of being overweight/obese were significantly 12.83 and 10.73 times higher among women aged 45–49 years in the unadjusted and adjusted analyses (Table 3). Marital status has a definite positive influence on body weight, as marital status is associated with underweight and overweight/obesity in both unadjusted and adjusted analyses. These findings revealed that ever-married women were 0.27 times less likely to be underweight compared to never-married women. When controlling for other variables used in the study, the findings revealed that ever-married women were 0.39 times less likely to be underweight, compared with never-married women. Similarly, ever-married women were found to be 2.62 times more likely to be overweight/obese compared to never-married women.

**Table 3:** Odd Ratios for socio-demographic factors associated with underweight and overweight/obese among weighted samples of women aged 15–49 years who had at least one live birth in the 5 years preceding the survey in South Africa, 2016.

**Socio-demographic factors**	**Underweight**		**Overweight/obese**	
	**UOR (95% CI)**	**AOR (95% CI)**	**UOR (95% CI)**	**AOR (95% CI)**
**Age**				
15–19	RC	RC	RC	RC
20–24	0.46 (0.28–0.74)^*^	0.49 (0.30–0.82)^*^	2.76 (2.15–3.54)^*^	2.60 (2.01–3.36)^*^
25–29	0.30 (0.17–0.51)^*^	0.38 (0.211–068)^*^	4.16 (3.24–5.35)^*^	3.62 (2.77–4.73)^*^
30–34	0.23 (0.12–0.44)^*^	0.34 (0.17–0.69)^*^	6.31 (4.81–8.29)^*^	5.00 (3.71–6.73)^*^
35–39	0.31 (0.17–0.56)^*^	0.46 (0.24–0.89)^*^	7.60 (5.70–10.13)^*^	6.30 (4.60–8.62)^*^
40–44	0.10 (0.03–0.27)^*^	0.15 (0.05–0.43)^*^	8.41 (6.22–11.38)^*^	6.79 (4.86–9.49)^*^
45–49	0.10 (0.04–0.28)^*^	0.16 (0.05–0.47)^*^	12.83 (9.19–17.93)^*^	10.73 (7.41–15.52)^*^
**Marital status**				
Never married	RC	RC	RC	RC
Ever married	0.27 (0.17–0.44)^*^	0.39 (0.23–0.68)^*^	2.62 (2.24–3.06)^*^	1.55 (1.28–1.86)^*^
**Population group**				
Black/African	RC	RC	RC	RC
Other	2.05 (1.32–3.19)^*^	2.65 (1.40–5.00)^*^	0.94 (0.75–1.18)^*^	0.59 (0.43–0.80)^*^
**Educational level**				
No education and primary	RC	RC	RC	RC
Secondary	1.15 (0.67–1.97)	0.76 (0.43–1.36)	1.04 (0.84–1.28)	1.61 (1.26–2.06)^*^
Higher	0.90 (0.40–2.02)	1.06 (0.43–2.59)	1.54 (1.12–2.11)^*^	1.41 (0.97–2.03)
**Residence**				
Urban	RC	RC	RC	RC
Rural	1.14 (0.81–1.61)	1.23 (0.75–2.02)	0.86 (0.74–0.99)^*^	1.11 (0.90–1.36)
**Work status**				
Not employed	RC	RC	RC	RC
Employed	0.41 (0.26–0.67)^*^	0.72 (0.43–1.22)	2.38 (2.02–2.81)^*^	1.33 (1.11–1.61)^*^
**Wealth quintile**				
Lowest	RC	RC	RC	RC
Middle	1.07 (0.71–1.63)	0.94 (0.59–1.49)	1.38 (1.15–1.65)^*^	1.48 (1.20–1.82)^*^
Highest	0.81 (0.53–1.22)	0.66 (0.38–1.13)	1.60 (1.36–1.89)^*^	1.80 (1.43–2.26)^*^
**Provinces**				
Western Cape	RC	RC	RC	RC
Eastern Cape	0.49 (0.18–1.34)	0.43 (0.14–1.28)	0.96 (0.65–1.40)	1.27 (0.82–1.99)
Northern Cape	2.18 (0.92–5.14)	1.58 (0.65–3.87)	0.64 (0.43–0.95)^*^	0.80 (0.52–1.23)
Free State	1.20 (0.49–2.96)	1.46 (0.54–3.93)	0.84 (0.57–1.25)	0.82 (0.52–1.29)
KwaZulu-Natal	0.45 (0.17–1.19)	0.35 (0.12–1.03)	0.84 (0.58–1.21)	1.20 (0.78–1.84)
North West	1.10 (0.44–2.73)	1.20 (0.44–3.32)	0.82 (0.56–1.22)	0.85 (0.54–1.34)
Gauteng	0.54 (0.19–1.58)	0.70 (0.22–2.18)	0.79 (0.53–1.18)	0.76 (0.48–1.20)
Mpumalanga	1.10 (0.46–2.64)	1.00 (0.37–2.71)	0.73 (0.50–1.06)	0.85 (0.55–1.33)
Limpopo	1.26 (0.53–2.99)	1.04 (0.38–2.86)	0.68 (0.47–0.99)^*^	0.85 (0.54–1.34)

* *Significant p-values: p < 0.005; p < 0.001; p < 0.0001 95% Confidence intervals (CI); AOR, adjusted odds ratio; UOR, unadjusted odds ratio; RC, Reference Category; Adjustment variables of the multivariable models are age, marital status, educational level, residence, work status, wealth quintile, and provinces*.

The adjusted analysis showed that ever-married women were 1.55 times more likely to be overweight/obese compared with never-married women (Table 3). Similarly, women who belong to the “other” population group were 2.05 times more likely to be underweight compared with Black/African women. Adjusting for other variables used in the study, women in the “other” population group were 2.56 times more likely to be underweight compared with the Black/African population group. With regards to overweight/obese, women in the “other” population group were 0.94 times less likely to be overweight/obese compared with women in the Black/African population group (Table 3). The unadjusted logistic regression analysis showed that women from the “other” population group were 0.59 times less likely to be overweight/obese compared with women in the Black/African population group. Further, there is a significant relationship between education and overweight/obesity, as women with higher education were 1.54 times more likely to be overweight/obese compared with women with no education or primary education. Women with secondary education were 1.61 times more likely to be overweight/obese compared to women with no education or primary education.

Conversely, rural women were found to be 0.86 times less likely to be overweight/obese compared to their urban counterparts, Employed women were 0.41 times less likely to be underweight compared to unemployed women in the unadjusted analysis (Table 3). This further indicated that employed women were 2.38 times more likely to be overweight/obese in the unadjusted analysis, and after adjusting for covariates, women who were employed were 1.33 times more likely to be overweight/obese compared to unemployed women (Table 3). As regards the wealth quintile, women in the middle and highest wealth quintiles were 1.38 and 1.60 times more likely to be overweight/obese, respectively. The adjusted regression analysis shows that women in the middle and rich wealth quintile were 1.48 and 1.80 times more likely to be overweight/obese, respectively. Furthermore, the province shows a statistical relationship with overweight/obesity in the unadjusted analysis. The findings show that women who reside in Northern Cape were more likely to be underweight compared to other provinces (UOR 2.18; 95% CI 0.92–5.14; AOR 1.58: 95% CI 0.65–3.87). Women residing in the Eastern Cape (AOR 1.27: 95% CI 0.82–1.99) and KwaZulu-Natal Provinces (AOR 1.20: 95% CI 0.78–1.84) were likely to be associated with overweight/obese body weight (Table 3).

## Discussion

In spite of several national and international efforts to improve the nutritional status of women of reproductive age, a substantial proportion of women are still plagued with underweight and overweight/obesity in South Africa. However, notable progress has been witnessed in the health delivery system along with the provision of the National Health Insurance Scheme (NHIS). South Africa needs to improve health awareness and public sensitisation about the health concerns of underweight and overweight/obesity to achieve the core targets of Sustainable Development Goal (SDG) 3.4 for the reduction of premature deaths from NCDs. The 2016 South Africa key indicator report revealed that the prevalence of overweight (27%) and obesity (41%) were highest among women in South Africa (32). This study established synchronicity of a 2-fold burden of underweight and overweight/obesity among women of reproductive age in South Africa, even though the prevalence of underweight was declining, yet overweight/obesity increased significantly in the period under study.

The study also observed that the key socio-demographic correlates of underweight were being aged 20–24 years, never married, in the “other” population group, having secondary education, rural, not employed, in the middle wealth quintile, and residing in Northern Cape Province. Since the data represent women of reproductive age (15–49 years) in South Africa, the findings of the study can be generalised to the general population in that age group. The study finding showed that there was a low prevalence of underweight among rural women in South Africa. This result correlates with other findings in sub-Saharan Africa, where underweight prevalence has decreased significantly (38–40). However, a few studies have reported the increased prevalence of underweight in both rural and urban areas in countries, such as Madagascar, Mali, and Senegal (41–43). With regard to overweight/obesity, the main socio-demographic correlates were increased, such as age (44–48), married, tertiary education, rural, employed, high wealth quintile, and residence in the Eastern Cape and KwaZulu-Natal provinces. These findings are consistent with previous studies conducted (41–43).

The high prevalence of overweight/obesity among women aged 15–49 years in South Africa showed differences when compared to the prevalence trends found in studies conducted in Asian countries, where the contrasting trend is observed. Several Asian studies have demonstrated that adult females were more likely to be underweight than overweight/obese (44, 49). The trends of body weight categories in South Africa in 2016 indicated that the prevalence of underweight is decreasing over the years while that of overweight/obesity is increasing. It has been observed that nutrition transition, changes in lifestyles, rapid urbanisation, increasing incomes, and consumption of high-fat food coupled with lack of physical activity are the key causes of the overweight/obesity epidemic in sub-Saharan Africa (31, 38), including South Africa (17, 43). For instance, a study conducted in Botswana reported that about 82% of people eat insufficient fruits and vegetables, 13% consume alcohol, 12% used tobacco, and about 52% of the respondents reported engaging in no or a low level of physical exercise (41, 42). In South Africa, about 59% of adults have reported consumption of fruits and vegetables while 49% reported that they consumed fruits only, and about 39% consume an unhealthy diet, and 8% and 5% engage in using tobacco products and risky drinking, respectively (14, 45). These findings are similar to evidence from countries, such as Nigeria (46), Ghana (47), Namibia (48), which are countries with an increasing prevalence of overweight/ obesity associated with poor lifestyles. These high prevalence rates of modifiable unhealthy lifestyles can aid in understanding the causes for increased rates of overweight/obesity in South Africa.

As in other parts of the world, it has been ascertained that high blood pressure and diabetes were more predominant among those who were overweight and obese in South Africa (12, 43). Risk factors, such as alcohol, tobacco use, physical inactivity, dietary intake, and sugary drinks, have been identified as high risks for overweight/obesity among the South African population; however, preventable behaviours could lead to its reduction (43). The study findings also demonstrated that women who are employed and are in the Black/African population group demonstrated a higher prevalence of overweight/obesity than women in the “other” population group, while women in the “other” population group were more likely to be associated with underweight than Black/Africans. These findings are similar to several extant studies conducted in South Africa (14, 43). Several scholars have shown a strong impact of socio-economic status on overweight/obesity, predominantly in women, causing disparities in their behaviours towards changes in their energy intake and expenditure, which, as a result, affect their body fat storage (50, 51). The racial disparity in overweight/obesity prevalence remained largely proportional for each respective educational level among Black/African women. The findings of this study reported that Black/African women in South Africa were more likely to be overweight/obese than women in the “other” population group. These outcomes are comparable to the study findings conducted in the United States of America, which illustrated racial trends associated with a higher prevalence of overweight/obesity among black women, such as African-American women (19, 20, 52).

Similarly, existing evidence from previous studies shows that South Africa has the highest proportion of people who were overweight and obese among Coloured (26%) and Black/African population groups (20%), with the majority being women (14, 35, 43). Hence, the advancement of social change, urbanisation, and ageing could be the possible reasons for the key drivers of the prevalence of overweight/obesity among Black/African women in South Africa. Thus, one of the preventive measures in reducing the health burden of overweight/obesity is by ensuring a greater focus on political will and regulation of the way in which products, such as sugar-sweetened drinks and other items, tobacco, and alcohol, have to be scrutinised in South Africa (14, 43). This study did not investigate the effects of these products and items on body weight, although they are generally believed to be linked with poor health outcomes. This study has identified several socio-demographic correlates of body weights of South African women of reproductive age. The findings from the multivariate analysis of this study have established that women were more likely to be overweight/obese with increased age (45–49 years), married, urban, with tertiary education, employed, in the highest wealth quintile, Black/African, and resident in the Eastern Cape and KwaZulu-Natal Provinces than underweight women. This finding is consistent with other studies conducted in India (44) and Nigeria (46).

A possible explanation for higher odds of women with higher education and high wealth quintile being overweight/obese might be due to lifestyles and dietary choices. Women with higher education may not associate their lifestyles with affluence, neglecting the health implication of overweight/obesity. In addition, women in the highest wealth quintile may be less physically active with better dietary choices, such as poor consumption of fruits and vegetables, a higher intake of highly caloric foods, and a poor routine of body exercise (40, 47). The 2016 SADHS report indicated that severe obesity increases with increasing wealth quintile for women. The multivariate analyses found out that rural women were more likely to be underweight compared to urban women. Urban women have quite a lot of advantages over their rural counterparts, such as higher levels of educational knowledge, greater awareness, employment, affluence, and easy access to health services, whereas rural women are often deprived of social and economic prospects (13, 46, 53). However, rural-urban has no significant relationship with body weight in the multivariate analysis. A study conducted in Tanzania reported a similar finding (54).

Our findings highlight the potential impacts of nutrition transition in rural areas as it requires urgent attention to fight against poverty, inequality, unemployment, and lack of basic social amenities. Our study has found that the two provinces having the highest levels of overweight/obesity were Eastern Cape, followed closely by KwaZulu-Natal, but this was not significantly associated in the adjusted model of overweight/obesity multivariate analyses. Contrary to the findings from the 2016 SADHS (32) and the General Household Survey (2016) (55), KwaZulu-Natal and Western Cape were the two provinces that had the highest overweight/obesity status (meanwhile, Western Cape was used in our study, as a reference category in the multivariate analysis). Although the explanations for these increasing trends are not completely understood, a few studies have reported that trends in overweight/obesity are not homogeneous across population strata, but they are defined by biological, socio-economic, and behavioural factors (9, 56–61). The identification of socio-economic and behavioural factors has an immediate prospect from a public health perspective, as these factors are potentially modifiable. In addition, the identification of biological factors is also of public health interest since this knowledge can help in targeting high-risk population groups more effectively, especially in grassroots communities, avoiding waste of resources associated with broad interventions. The presence of socio-economic inequalities in overweight/obesity prevalence is a well-established finding and has been previously confirmed among the South African population (10, 62).

### Limitations of the Study

Since this study is based on cross-sectional data, no causal relationships can be deduced from this type of data. However, the ORs were used to determine how different socio-demographic factors are risk factors for either underweight or overweight/obesity. One of the major limitations is that some of the key modifiable factors associated with nutritional transition were excluded from the analysis because the datasets used did not cover behavioural subject themes, such as lifestyle variables. This study is constrained to socio-demographic factors and as such one cannot explain the behavioural aspects of underweight and overweight/obesity. Women who did not respond to BMI questions or who were pregnant at the time of the survey were excluded. In addition, only weighted datasets for women 15–49 years who had at least one live birth in the 5 years preceding the survey were included in this study analysis. Despite the above limitations, the present study uses nationally representative data on underweight and overweight/obesity in South Africa to present generalizable findings.

## Conclusion

The present study shows that the prevalences of underweight and overweight/obesity were 4.1 and 61.6%, respectively, among women in South Africa. However, the 2016 SADHS key indicators revealed a prevalence of 3.0% for underweight and 68.0% for overweight/obesity among women in South Africa (32). Key socio-demographic factors connected with underweight included women who reside in rural areas and belong to “other” population groups, while the factors linked with overweight/obesity were increasing age, Black/African, higher educational attainment, and higher wealth quintile. Women who were formally employed were less likely to be underweight. Locally relevant policy and interventions should not only target improvement in the socio-economic status of South African women but should also focus on the education of women around the benefits of regular physical activity and healthly dietary choices. It is, therefore, important to take cognizance of those direct interventions, which are designed to tackle the health burden of underweight and overweight/obesity to alleviate health problems associated with nutrition transition. Further research is needed to unravel other factors accompanying underweight and overweight/obesity, which were not enclosed in the existing SADHS datasets. It is also crucial to explore the underlying behavioural factors for underweight and overweight/obesity, such as reasons for low dietary intake, excessive alcohol intake, and tobacco use.

## Data Availability Statement

The datasets analysed during the current study are available from the DHS Program: https://dhsprogram.com/data/available-datasets.cfm.

## Ethics Statement

The studies involving human participants were reviewed and approved by Ethical review and approval for procedures and questionnaires for standard DHS surveys are provided by ICF Institutional Review Board (IRB). Country-specific DHS survey protocols are reviewed by the ICF IRB and typically by an IRB in the host country. Written informed consent to participate in this study was provided by the participants' legal guardian/next of kin.

## Author Contributions

MA wrote, analysed the DHS datasets, interpreted the DHS data, and was a primary contributor in the manuscript writing. MA and EI revised the manuscript and agreed on the findings and the views expressed within. All authors read and approved the final manuscript.

## Funding

MA received funding for this research from the North-West University Post-doctoral Research Fellowship (PDRF), Mafikeng, South Africa (Postdoc Grant No. NWU PDRF Fund NW. 1G01487). All funders have been credited and all grant numbers were correctly included in this section of Funding.

## Conflict of Interest

The authors declare that the research was conducted in the absence of any commercial or financial relationships that could be construed as a potential conflict of interest.

## Publisher's Note

All claims expressed in this article are solely those of the authors and do not necessarily represent those of their affiliated organizations, or those of the publisher, the editors and the reviewers. Any product that may be evaluated in this article, or claim that may be made by its manufacturer, is not guaranteed or endorsed by the publisher.

## Abbreviations

AIDS: Acquired Immunodeficiency Syndrome
AOR: Adjusted odds ratio
BMI: Body Mass Index
CI: confidence interval
DHS: Demographic Health Survey
DUs: Dwelling units
HIV: Human Immunodeficiency Virus
NHIS: National Health Insurance Scheme
NCDs: Non-communicable Diseases
OR: Odds ratio
PSUs: Primary sampling units
SDG: Sustainable Development Goal
SES: Socio-economic status
SSA: sub-Saharan Africa
UOR: Unadjusted odds ratio
WHO: World Health Organisation.
